# Supplementary material for: Chromothripsis during telomere crisis is independent of NHEJ, and consistent with a replicative origin
Source: Genome Res. 2019 May;29(5):737–49. doi: 10.1101/gr.240705.118 (PMC6499312; doi:10.1101/gr.240705.118)
Supplement: Supplemental Material [file supp_gr.240705.118_Supplemental_file_1.zip › contigs/annotated_contigs/DB53/contig.2.DB53_length_620_mean_cov_6.53225806452.docx]

**DB53_length_620_mean_cov_6.53225806452**

```
CTTAAACAACTAACCAAACCACACATGCAGAAAAATACTGCTAAAAGGCAAATCATTATATTGACATCAACATGATAATAATATCAGAA
 >chr5:80384586-80384859 - E=1e-152 p=2e-02
ATATTCATAGCTGACAGAAGTGCCTTAAACATGTGGTACATAACAAGGAATTAATAACATATGGGCATGAATAAAGGGAGAAATTGTTC

ATGATTGTTTTCTCTCCAATTGCAGTGAAAAAAAATGACATTCAGTATTTGACCTTCCAGCATTTTTCATTATTGAATGTTTTTTCAAG

CTCAG|A|TTGTAGATTTAAAGAATGGCAGTTATAGCAAAAACCTCCTTTTCCTCCCCAATTGCCTAATCAACATTTAGGGCCCCCTCC
      >chr5:80527565-80527913 - E=1e-197
ACTCACCCGACCTCAACACCTTATATTTTATAGAGTAAATGTTTATGTTAGTAAGGTCAGAAGAAAAATTGGGTCATTGTTGAGAAAAG

AGGGTACCACCCTTCTTTTGGGGGACTGGTCCTCTTCAAGCTTATTATAAAGGTGGTGTGATGGGGATGAAATCTCTACAAGGGACTCA

CAGTAGAAATTCTGGCTTTCTGGAAATCTTTCAAATGCCATCCCTGTCACATGGCATCTATTCCCTTCTCATCGTTACCAACTCCTTT
```
